# A Composite Hydrogel Based on Pectin/Cellulose via Chemical Cross-Linking for Hemorrhage

**DOI:** 10.3389/fbioe.2020.627351

**Published:** 2021-02-02

**Authors:** Wancheng Chen, Sijie Yuan, Jie Shen, Yongsheng Chen, Yang Xiao

**Affiliations:** ^1^Translational Medicine Center, Jiangmen Central Hospital, Jiangmen, China; ^2^Department of Endocrinology and Metabolism, The Third Affiliated Hospital, Southern Medical University, Guangzhou, China; ^3^Department of Food Science and Engineering, Jinan University, Guangzhou, China

**Keywords:** pectin, cellulose, cross-linking, hydrogel, hemostasis

## Abstract

Hydrogel-based material have been demonstrated promising potential for hemostasis. Herein, we prepared a composite hydrogel (CH-P 40%) by combining pectin and cellulose in ionic liquid. The superficial morphology of the CH-P 40% was explored by SEM; the internal chemical bonds, crystal form and thermal stability were determined via FTIR, XRD and thermogravimetric analysis, respectively. The biocompatibilities of the CH-P 40% hydrogel was evaluated by MTT, flow cytometry, and histological observation with H&E staining. Furthermore, the hemostatic effect was evaluated via the blood clotting index and mouse liver hemostatic model. The results showed that the CH-P 40% hydrogel exhibited a dense network structure and retained its chemical bonds, including the OH, CH, C=O, -CH2, CO, C1-H, and β-glycosidic bonds. Simultaneously, the hydrogel retained the Cellulose I and II crystal structure and favorable thermal stability. Moreover, the proliferation rates of CH-P 40%-treated cells increased (*P* > 0.05), and there were no pathological lesions in the mouse organs, which suggests favorable biocompatibility. The results showed less bleeding in the hydrogel-treated liver wound within 3 min. Overall, the pectin-cellulose hydrogel is stable and possesses favorable biocompatibility and hemostatic ability, further highlighting that the composite hydrogel has the potential to be rapid hemostatic biomedical material.

## Introduction

Hemorrhage is a common symptom in surgery and the military and result in serious complications; moreover, uncontrolled hemorrhage even leads to death (Zhao et al., 2018). Thus, it is essential to develop effective hemostatic materials. In addition to exhibiting basic mechanical properties and biocompatibility, a superior dressing should exhibit favorable swelling and non-allergy properties and skin recovery (Jia et al., 2018, 2019). Furthermore, it stimulates the production of lipid to improve skin barrier function, thus inhibiting inflammatory response (Li et al., 2017a; Zhou et al., 2018, 2020b). Currently, hemostatic materials applied in clinical include biological and synthetic materials, such as rubber, regenerated oxidized cellulose, biological adhesives, composite chitosan-based wound dressing, and hydrogels (Hickman et al., 2018; Huang et al., 2020). Hydrogels possess the superior properties for hemostasis, including flexibility, antibacterial activity, degradability (Gando, 2015; Leonhardt et al., 2019).

As the natural hydrogels, polysaccharide biopolymers have become increasingly popular in the preparation of hemostatic agents. They form a network structure with favorable swelling properties and several properties common to living tissues (Chen et al., 2020). To the best of our knowledge, cellulose- and pectin- hydrogels have been extensively investigated (Kabir et al., 2018; Kowalski et al., 2019).

Cellulose hydrogels have been considered beneficial as a biocompatible medical material for the following reasons (Van De Ven and Sheikhi, 2016; Curvello et al., 2019; Zhang et al., 2020): (1) Cellulose's multiple hydrogen bonds and domains form a dense network with certain rigidity and cross-linking, suggesting the advantage of swelling. (2) The network structure of cellulose leads to good mechanical character and cell scaffolding capabilities. (3) Naturally derived cellulose is not immunogenic and is easily biodegradable. Because of these characteristics, cellulose-based hydrogels are commonly used as surgical hemostatic agents, such as diabetic foot and/or wound dressings (Gagnon et al., 2017; Bonnin et al., 2019). However, even though oxidized regenerated cellulose is reabsorbable, it is still common for postoperative abscess, tumors, or hematoma to develop (Behbehani and Tulandi, 2013; Piozzi et al., 2018), which further limits the use of cellulose hydrogels for hemostasis. The microcrystalline cellulose used here is partially depolymerized cellulose synthesized from α-cellulose, which was obtained from fibrous plant material (Shlieout et al., 2002). Pectin, a linear polysaccharide polymer, is derived mainliy from juice industry. It was environmentally friendly and easily degraded, suggesting ideal biocompatibility and biosecurity (Douglas et al., 2019). Due to the quintessential structure, pectin was used as the blend to generate a composite material in different fields (Thakur, 2019). Moreover, most natural pectin exerts anti-inflammatory and antioxidant activity because of the esterified galacturonic acid units, thus facilitating its clinical application (Li et al., 2017b; Mzoughi et al., 2018; Han et al., 2019). However, pectin probably loses its integrity and mechanical properties under physiologic conditions.

**Figure 1:**
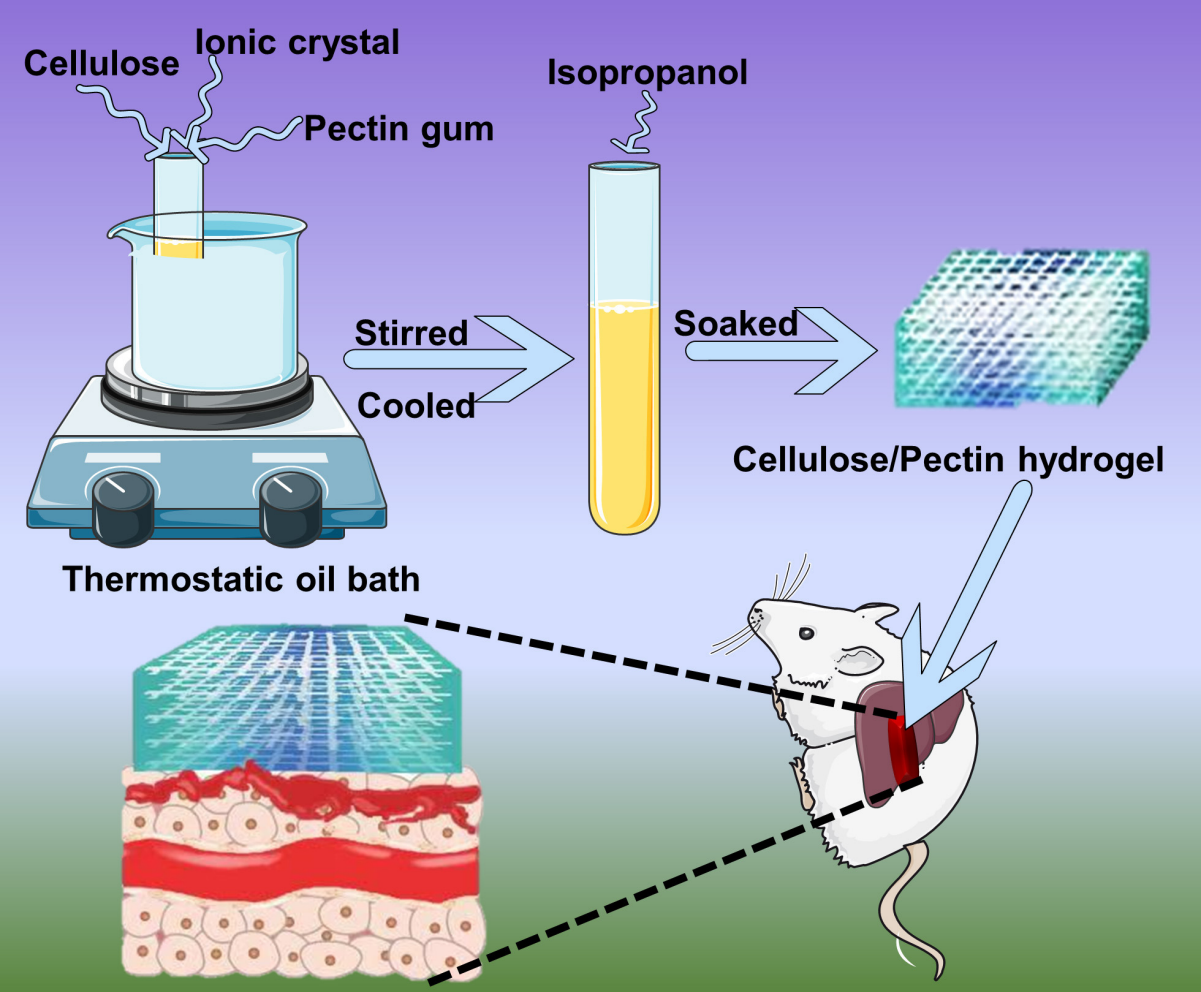
Schematic illustration of cellulose/pectin hydrogel for hemostasis.

Here, we proposed mixing pectin and cellulose in various proportion crosslinked by ionic liquid [AMIm]Cl to prepare a composite hydrogel that maintains the advantages of both for hemostasis (Kadokawa et al., 2009). As reported, chemical cross-links was widely applied in hydrogels modification and was essential to the static elasticity (Yoshimura et al., 2005; Kari, 2020). The present study (Figure 1) was performed to (1) prepare a hemostatic agent with cellulose and pectin; (2) characterize the prepared composite hydrogel using fourier transform infrared spectroscopy (FTIR), X-ray diffraction (XRD), thermo gravimetric analysis (TGA), and scanning electron microscopy (SEM); (3) analyze the biocompatibilities using methyl thiazolyl tetrazolium test (MTT test), apoptosis, and necrosis rate; (4) evaluate the therapeutic ability for liver injury hemorrhage *in vivo*.

## Materials and Methods

### Materials

Microcrystalline cellulose was purchased from PanEra (Guangzhou, China). 1-Allyl-3-methylimidazolium chloride ([AMim]Cl), pectin and methylene blue were obtained from Aladdin (Shanghai, China). DMEM and fetal bovine serum (FBS) were from Gibco (America). Other chemical reagents employed in the study were of analytical grade.

### Preparation of Composite Hydrogel

The composite hydrogel was synthesized according to a previous reported procedure (Kadokawa et al., 2009). Firstly, 0.5 g of microcrystalline cellulose was added to 10 g of ionic liquid [AMIm]Cl and stirred for 5 h at 100°C until dissolved. Additionally, pectin was added to the test tube as listed in Table 1 and stirred 3 h at 100°C. The materials were completely dissolved, cooled to room temperature and poured into a petri dish to prepare the coarse hydrogel. Finally, the coarse hydrogel was soaked in an isopropanol-water (1:1) solution to fully remove ionic liquid, thoroughly soaked and cleaned by deionized water and freeze-dried to yield the pure cellulose-pectin hydrogel (CH-P) for the next experiment.

**Table 1:** Formula of the samples.

**Sample**	**Cellulose (g)**	**Pectin (g)**	**[AMIm]Cl (g)**
CH-P20%	0.5	0.1	10
CH-P40%	0.5	0.2	10

*CH-P 20%: The weight ratio of pectin and cellulose is 5:1. CH-P 40%: The weight ratio of pectin and cellulose is 5:2*.

### Characterization of Composite Hydrogel

The surface morphology characteristics of CH-P were observed by scanning electron microscope (SEM, S-3700N, Hitachi, Japan) after gold deposition. Infrared spectra was analyzed on fourier transform infrared spectroscopy (FTIR, VERTEX-33, Bruker, Germany) from 4,000 cm-1 to 400 cm-1 and the crystal forms were delineated using an X-ray diffractometer (D8 ADVANCE, Bruker, Germany) in the 5~60° scanning angle range. Thermal stability was examined by thermogravimetric analysis at 30–600°C with 20°C/min heating rate by Thermo gravimetric analysis (TGA, TGA-Q500, TA Instruments, America).

### Cell Culture

The biocompatibility of the composite hydrogel was evaluated with RAW 264.7 and 3T3 cells. Cells were cultured on 6-well or 96-well plates in DMEM with 10% FBS and 1% penicillin-streptomycin at 37°C with 5% CO2.

### Cell Viability Evaluation

CH-P was soaked with 75% ethanol in 96-well plates for 24 h. RAW 264.7 or 3T3 cells were seeded on the hydrogel for 24 h. Then, cultured medium was exchanged with 200 μL methyl thiazolyl tetrazolium (MTT, 0.5 mg/mL) serum-free medium. After 4 h, the supernatant was removed, and 150 μL/well DMSO was added. The plate was shaking for 10 min, and the absorbance was recorded at 490 nm by a microplate Reader. The cell viability representing the biocompatibility of hydrogel was calculated by formula below:

Cell viability (%) = (OD sample–OD blank)/(OD control–OD blank) × 100%.

The rate of apoptosis and necrosis of RAW 264.7 and 3T3 cells were detected flow cytometry with Annexin V-FITC/PI detection kit (BD LSRFortessa TM, New York, America).

### Evaluation of Blood Clotting Property

The coagulation property of the composite hydrogel was evaluated by the blood clotting index as previous reports. Here, the smaller value of blood clotting index suggested the better coagulation effect (Zhang et al., 2019). Briefly, a total volume of 100 μL of fresh blood was added to a test tube and incubated with a piece of CH-P 40% for 5 min. Then, the blood clotting indexes of each group were obtained by measuring the absorbance at 545 nm. The specific formula is as follows:

Blood clotting index (%) = A sample / A control × 100%, where A sample and A control represent the absorbance of the CH-P 40% hydrogel group and the control group, respectively.

### Evaluation of Hemostasis *in vivo*

C57BL/6 mice were obtained from Guangdong Medical Laboratory Animal Center, and the experimental protocol was approved by the Ethics Committee of Southern Medical University. Eight male C57BL/6 mice aged 8–12 weeks were randomly assigned into the treated group or the control group. After anesthesia, a liver hemorrhage model was constructed by performing abdominal operations with 2 mm needle. Then, the wound of each mouse was covered by CH-P 40% hydrogel gauze (treated group) or a single piece of gauze (control group).

### Statistical Analysis

All the results were recorded as the mean ± SD. Two-tailed Student's *t*-test was used to analyze the differences between two groups. One-way ANOVA with multiple comparison was used to analyze the differences between three or more groups. *P* < 0.05 was indicated as significant difference.

## Result and Discussion

### The Surface Morphology of Composite Hydrogel

**Figure 2:**
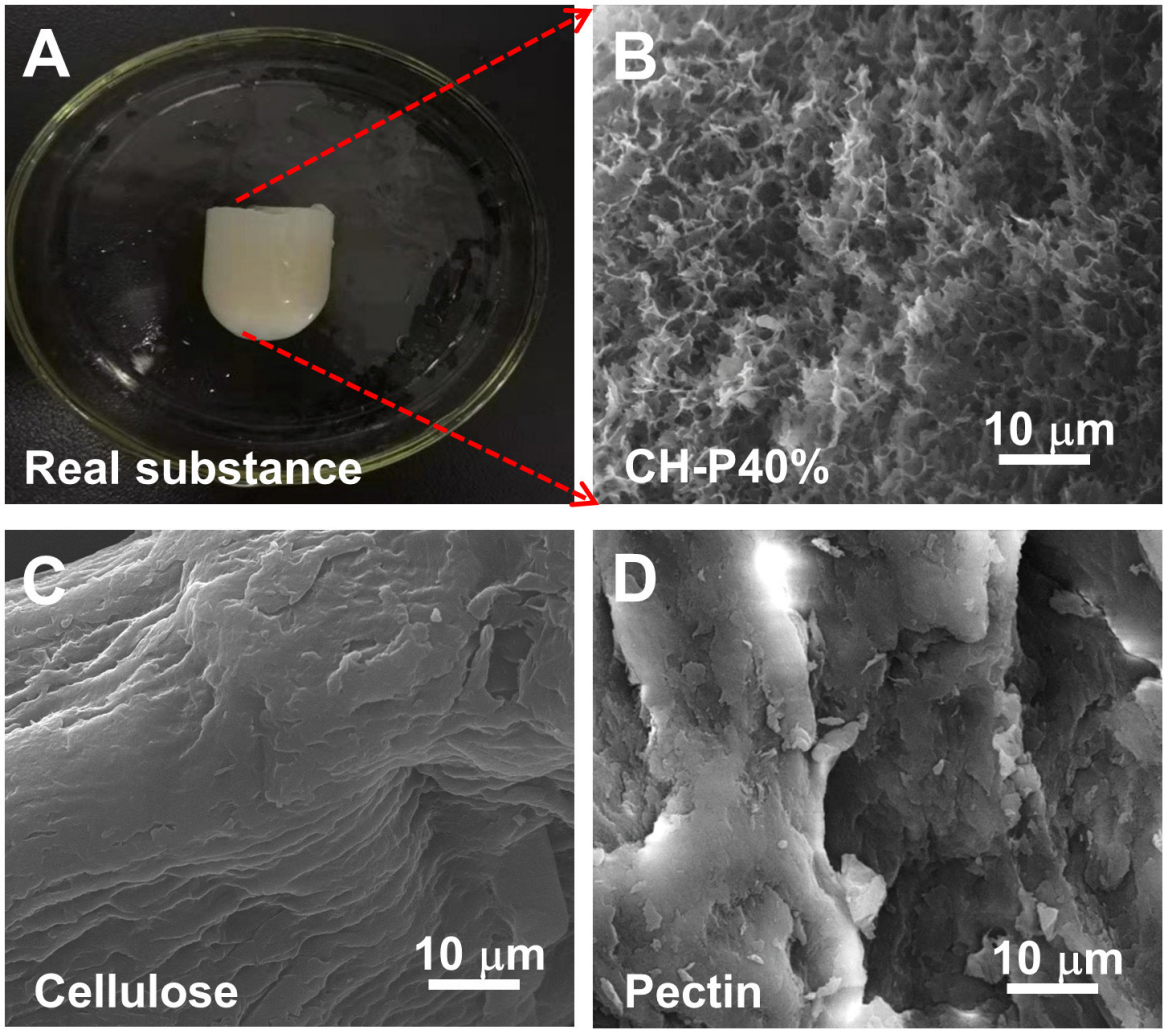
The surface morphology characteristics of the as-prepared hydrogel. **(A)** Optical image of composite hydrogel, **(B–D)** SEM image of composite hydrogel, cellulose hydrogel, and pectin hydrogel.

The external macrostructural characteristics of the hydrogel were explored via SEM. As shown in Figure 2, CH-P showed more folds on the surface (Figure 2B); however, cellulose (Figure 2C) and pectin (Figure 2D) showed irregular block-like structure with smooth surfaces, but without porous structure. The result indicated that CH-P had a spongy and porous feature. Notably, CH-P contained a filamentous network structure, which indicated an increased superficial area and greater capacity to absorb aqueous solution and accelerate the formation of blood clots. On the other hand, the denser network structure within CH-P empowers the cells to grow inside and protects wounds from the bacteria. Taken together, these macrostructural characteristics suggested that CH-P may be an outstanding wound dressing.

### Physicochemical Properties of Composite Hydrogel

**Figure 3:**
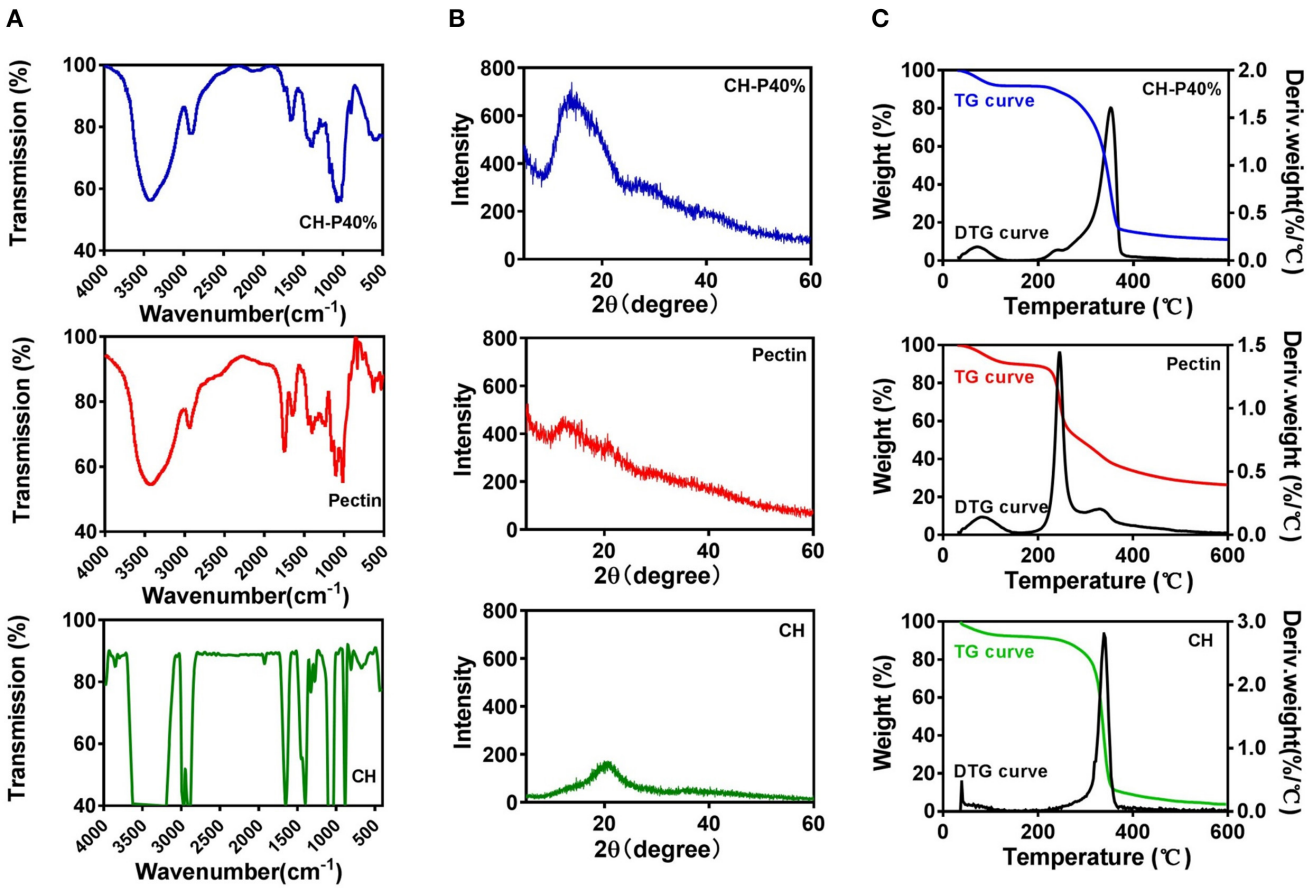
Characteristics of cellulose (CH) hydrogel, pectin hydrogel and CH-P40% hydrogel. **(A)** FTIR, **(B)** XRD, **(C)** TG and DTG.

To further investigate the physical performance of the composite hydrogel, FTIR and XRD experiments were performed. The FTIR spectrum in the wavelength range of 500–4,000 cm^−1^ (Figure 3A) showed that cellulose possessed a narrow and sharp peak at 3,423 and 2,922 cm^−1^ owing to the O-H and C-H in methyl and methylene groups, respectively. The absorption peak at 1,654, 1,401, and 1,159 cm^−1^ indicated the presence of C=O, -CH2, and C-O respectively, which were considered as the characteristic absorption peaks of cellulose. The absorption peaks at 1,069 and 897 cm^−1^ were attributed to the presence of C-O and the vibrational frequency of C1-H as well as β-glycosidic bonding, which were consistent with the pervious findings (Trombino et al., 2019). For pectin, the absorption peak at ~2,935 cm^−1^ suggested the presence of C-H, including the stretching and bending vibration of CH, CH2, and CH3. The frequency peak values of 1,015, 1,235, 1,103, and 1,443 cm^−1^ represent the extension and stretching of C-O, C=O and the asymmetric stretching mode of CH3, respectively (Willats et al., 2001). As shown in infrared spectra, the CH-P 40% hydrogel presented characteristic peaks similar to those of cellulose and pectin (Figure 3A), suggesting that the composite hydrogel maintained the good biological activity as cellulose.

As shown in Figure 3B, the diffraction peaks were close to 2θ = 13.8° and 2θ = 22.1°, which corresponded to cellulose type I and II and were consistent with the characteristic peaks of the cellulose crystal. However, there was no obvious characteristic peaks for pectin, which indicated pectin may lack a crystallization region in the internal structure. The X-ray diffraction pattern of CH-P 40% hydrogel was closed to 2θ = 13.4°, in accordance with cellulose hydrogel, indicating that the properties of the composite hydrogel were more similar to those of cellulose. The stage of crystallinity is related to the thermal stability, resulting in a more ordered structure and increased heat resistance of the hydrogel (Segal et al., 1959).

Thermo gravimetric analysis (TGA) and derivative thermogravimetry (DTG) were applied to test the stability of hydrogels (1) to examine the effect of human temperature stimulation on the stability of hydrogels through TGA curves and (2) to evaluate the effect of pectin on the stability of the cellulose hydrogel. As shown in Figure 3C, the first weight loss plateau appeared at a temperature below 100°C and was caused by the water evaporation of the samples, while the second weight loss plateau was mainly caused by thermal decomposition of the samples and appeared in the range of 200–400°C. In the first stage, there was no loss of mass in the CH-P 40% hydrogel during the heating process from 30 to 50°C, indicating that the hydrogel possibly remained stable during the body temperature stimulation. Moreover, according to the TGA curve, the thermal stability of the CH-P 40% hydrogel was slightly improved becasue of pectin. As shown in DTG curves, the highest peaks of pectin and cellulose appeared at about ~245 and 341°C, and their weights were ~1.4 and 1.3%/°C, respectively. Compared with pectin and cellulose, the CH-P 40% hydrogel presented a very weak peak at approximately 72°C and a sharp peak ~353°C, in accordance with weights of ~0.14 and 1.61%/°C. The addition of 40% pectin increased the decomposition temperature, suggesting that pectin could slightly improve the thermal stability of the prepared hydrogel.

Thus, the synthetic method using 1-allyl-3-methylimidazolium chloride could retain the functional groups and chemical bonds of cellulose and pectin to maintain the traits of individual components. Therefore, the ideal mechanical properties could suggest that CH-P may be a reliable wound dressing.

### Biocompatibility of the Composite Hydrogel

**Figure 4:**
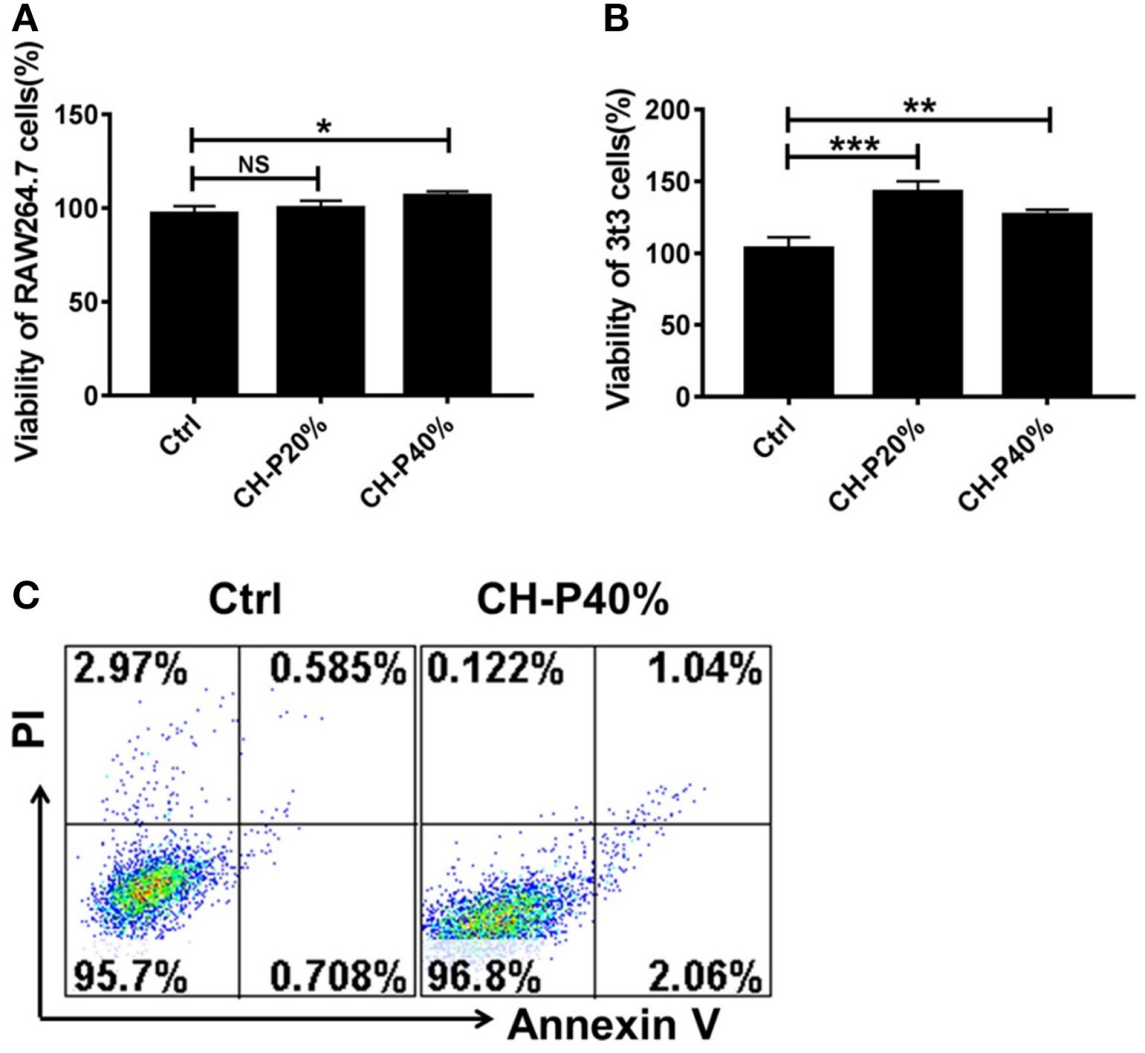
Toxicity evaluation of the prepared hydrogel. **(A,B)** Cytotoxicity of composite hydrogel to RAW 246.7 cells and 3T3 cells detected by MTT (*n* = 3), **(C)** Cytotoxicity of composite hydrogel to 3T3 cells detected by Annexin V/PI kit (*n* = 3). *p < 0.05, **p < 0.01, ***p < 0.001 vs. control.

**Figure 5:**
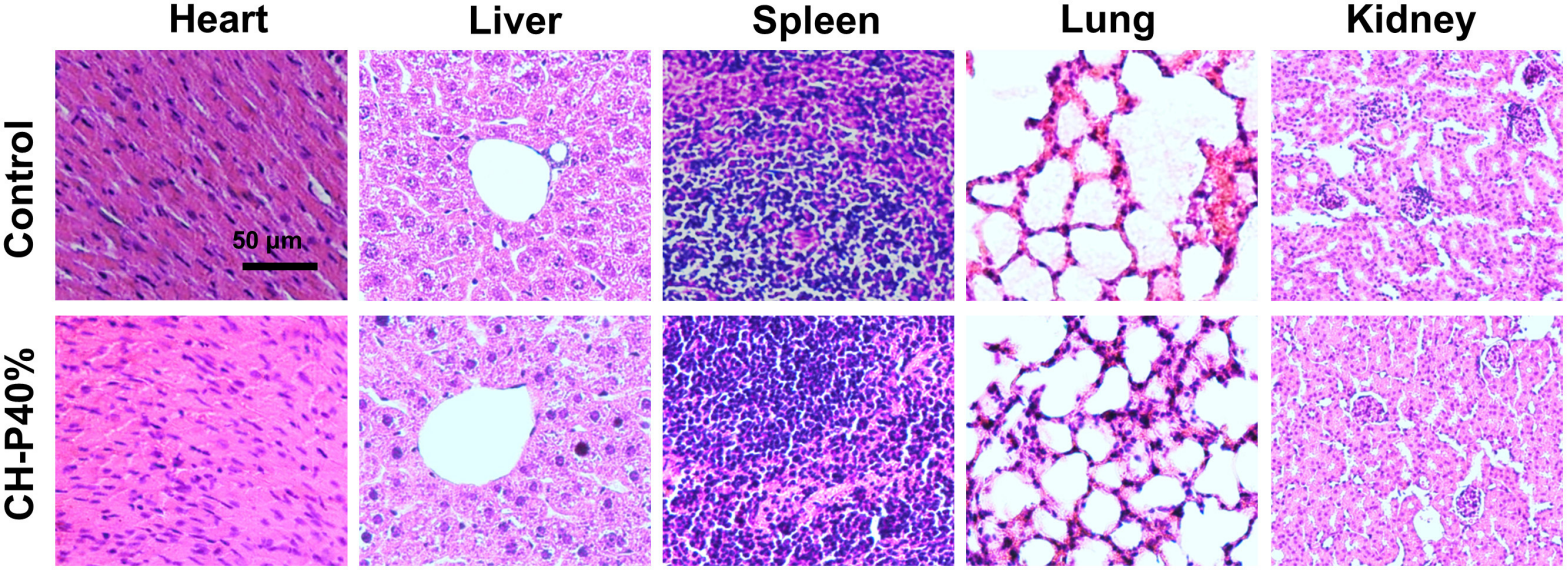
Pathological section of animal organs (*n* = 4). Values were expressed as means ± SD. One-way ANOVA was used to compare the difference.

To evalute the biocompatibility of the CH-P 40% hydrogel, we conducted experiments *in vitro* and *in vivo*. The morphologies of CH-P 40% hydrogel-treated RAW 264.7 and 3T3 cells hardly changed, maintaining a spindle shape and adherence to the wall. On the other hand, the cell proliferation rates of CH-P 20%-treated and CH-P 40%-treated RAW 246.7 cells and 3T3 cells were enhanced compared with those of the control group (*P* < 0.05), suggesting CH-P could induce better cell proliferation (Figures 4A,B). Moreover, the survival rate of CH-P-treated 3T3 cells was more than 95%, as shown in Figure 4C, suggesting that CH-P has excellent biocompatibility. In addition, immune compatibility was correlated with the biosafety of CH-P. RAW 264.7 cells, as candidates for macrophages directly contacting hydrogel dressing (Rocca et al., 2016), were indicated to proliferate well on CH-P 40%. These results indicated that CH-P 40% hydrogel possessed favorable immune compatibility to avoid inducing macrophages differentiation. As shown in Figure 5, no obvious abnormalities were observed in the major organs (including heart, liver, spleen, lung, kidney) in the mice for 10 days while their liver wounds covered with the CH-P 40% hydrogel, suggesting that the hydrogel was non-toxic to mice.

### Hemostasis Performance of Composite Hydrogel

**Figure 6:**
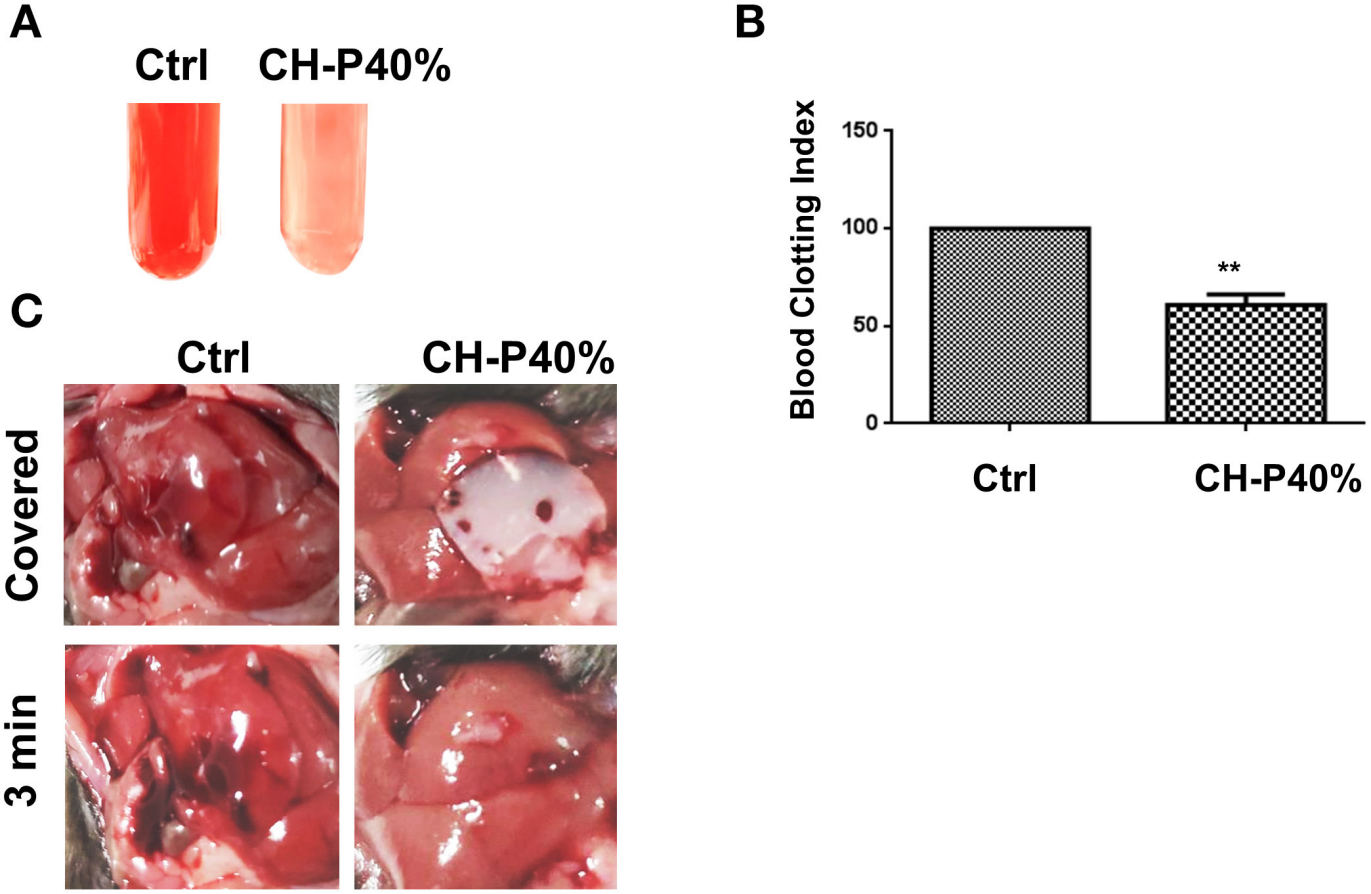
Evaluation of hemostatic effect of the prepared hydrogel. **(A)** Blood agglutination picture (*n* = 3); **(B)** Blood Clot Index (BCI) (*n* = 3); **(C)** Liver hemostasis experiment (*n* = 4). Values were expressed as means ± SD. One-way ANOVA was used to compare the difference. **p < 0.01 vs. control.

**Figure 7:**
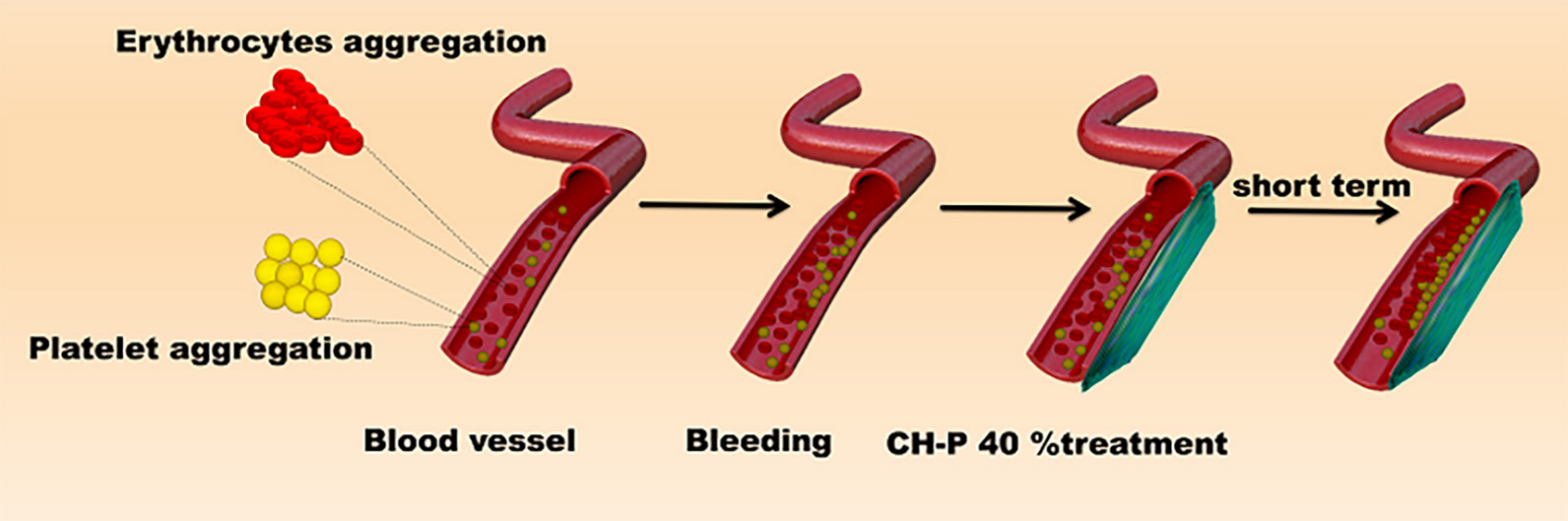
Schematic diagram of hemostatic mechanism.

Since CH-P has good characteristics required for a hemostatic agent, we evaluated the hemostatic effect of the sutured hydrogel *in vitro* and *in vivo*. The blood-clotting capability of CH-P 40% was assessed by the Blood Clot Index (BCI), which is negatively correlated with hemostasis capacity *in vitro* (Wang et al., 2019). The CH-P 40%-treated group had a lighter solution color and lower BCI than the control group (Figures 6A,B). Subsequently, the hemostasis capacity was evaluated according to the volume of hemorrhage in 3 min with employment of hepatic hemorrhage model. The results suggested that CH-P 40% could more effectively stop the blood loss (Figure 6C), probably due to the endogenous coagulation followed by rapidly sealed filling and external covering of the bleeding tissue. The underlying mechanism was as follows (Figure 7): erythrocyte aggregation and platelet aggregation occurred upon bleeding, and the hydrogel accelerated the process by quickly filling to form a barrier to prevent leakage of the tissue fluid and cells (Wang et al., 2019). In addition, the nanostructures inside the hydrogels could not only absorb percolate but also activate platelets to promote hemostasis (Saini et al., 2016). At the same time, its effects on skin lipids and skin barrier recovery need further study (Cui et al., 2018; Zhou et al., 2020a).

## Conclusion

The present research demonstrated a natural composite hydrogel synthesized by combining flexible pectin and cellulose in ionic liquid. This composite hydrogel exhibited a dense network structure and outstanding thermal stability. Additionally, the composite hydrogel showed no effects on cell proliferation and had favorable properties in liver hemostasis. The composite hydrogel may have potential as a hemostatic biomedical materials.

## Data Availability Statement

The original contributions presented in the study are included in the article/supplementary material, further inquiries can be directed to the corresponding authors.

## Ethics Statement

The animal study was reviewed and approved by Ethics Committee of Southern Medical University.

## Author Contributions

All authors listed have made a substantial, direct and intellectual contribution to the work, and approved it for publication.

## Conflict of Interest

The authors declare that the research was conducted in the absence of any commercial or financial relationships that could be construed as a potential conflict of interest.
